# A pilot study to establish feasibility and acceptability of a yoga and self-management education intervention to support caregivers and care receivers with persistent pain

**DOI:** 10.3389/fresc.2024.1397220

**Published:** 2024-10-03

**Authors:** Arlene A. Schmid, Christine A. Fruhauf, Aimee L. Fox, Julia L. Sharp, Jennifer Dickman Portz, Heather J. Leach, Marieke Van Puymbroeck

**Affiliations:** ^1^ Department of Occupational Therapy, Colorado State University, Fort Collins, CO, United States; ^2^ Department of Human Development and Family Studies, Colorado State University, Fort Collins, CO, United States; ^3^ Family Science, Utah Valley University, Orem, UT, United States; ^4^ School of Medicine, University of Colorado Anschutz Medical Campus, Aurora, CO, United States; ^5^ Department of Health and Exercise Science, Colorado State University, Fort Collins, CO, United States; ^6^ Department of Parks, Recreation, and Tourism Management and Graduate School, Clemson University, Clemson, SC, United States

**Keywords:** persistent pain, pain management, yoga, self-management education, health management, caregiving dyad, telerehabilitation, rehabilitation

## Abstract

**Introduction:**

Approximately 75% of caregivers providing unpaid care to family members or friends experience persistent pain. Simultaneously, individuals who require caregiving commonly experience pain. The inherent complexity of pain is enhanced by relationship dynamics of two closely tied individuals (i.e., caregiving dyad = caregivers and care recipients). Currently there are no proven pain interventions that target the caregiving dyad. Thus, the purpose of this pilot study was to assess the feasibility of a new behavioral multi-modal intervention, the Merging Yoga and self-management to develop Skills (MY-Skills) intervention.

**Methods:**

Each participant was part of a caregiving dyad and all participants had moderate to severe musculoskeletal pain, a score of ≥4 of 6 on the short mini-mental status exam, were ≥18 years old, sedentary, able to speak English, able to stand, and living at home. Participants were randomized to MY-Skills or the control group. MY-Skills was offered twice a week for eight weeks and each two-hour session included yoga and self-management education developed specifically for caregiving dyads experiencing persistent pain. MY-Skills was group based and developed as an in-person intervention. Due to Covid-19, the intervention was moved online and data are presented for in-person and online cohorts. Benchmarks for feasibility were set *a priori*, addressing: recruitment, attrition, attendance, safety, acceptability/satisfaction, and study completion.

**Results:**

Thirteen participants completed the in-person MY-Skills intervention (caregivers *n* = 7, care-receivers *n* = 6) and 18 individuals completed the online MY-Skills intervention (9 dyads). Most participants had pain for ≥10 years. Recruitment and attrition benchmarks for the in-person intervention were not met; yet they were met for the online version. In-person and online MY-Skills intervention attendance, safety, acceptability/satisfaction, and completion exceeded benchmark criteria.

**Discussion:**

The MY-Skills intervention appears feasible and acceptable, however changes to recruitment criteria are necessary. Additional testing and larger sample sizes are required to test efficacy.

**Trial registration:**

Clinicaltrials.gov, #NCT03440320.

## 1 Introduction

Chronic, or persistent pain, is a global issue which negatively impacts individual's biological, psychological, and social functioning with subsequent economic impacts (1, 2). The negative impact may affect quality of life (QoL) and performance and participation in life activities (3–6). While persistent pain is a global issue, it is of note that over 100 million Americans are thought to have persistent pain (7), which is pain that lasts more than three to six months.

Persistent pain can be complex, stemming from trauma or illness, and often explained by the Biopsychosocial Model. The Biopsychosocial Model supports a holistic approach to rehabilitation and suggests that causes and outcomes of chronic disease, such as pain, commonly involve the interaction of multiple factors, including biological, psychological, and social-environmental factors (8). Thus, due to the complexity of persistent pain and necessity of addressing the factors identified in the Biopsychosocial Model, it is thought that comprehensive multi-modal interventions that address the whole person are necessary for successful pain and health management (7, 9, 10).

Since persistent pain is commonly linked to disability and disease, often a need for caregiving is indicated. Nearly 66 million Americans identify as family caregivers with 74% of caregivers reporting pain (11). Other deleterious effects related to caregiving include risk of injury, caregiver burden, depression and ultimately worse QoL and increased death rates (12–14). While not well studied, persistent pain may be experienced by the care recipient and caregiver (i.e., caregiving dyad), and the pain is likely a complex interplay of biological, psychological, and social conditions that impact the caregiving relationship (8, 15). It is believed that the use of interventions that target both members of the caregiving dyad are useful and beneficial (15). For example, dyadic interventions are feasible and improve outcomes after dementia, cancer, or stroke (16–20), yet thus far, programs for individuals with chronic pain are limited to spousal-only dyads (21, 22). Enhanced outcomes from dyadic interventions may be related to the inclusion of both individuals in the dyad, potentially improving: intervention adherence and attendance (16), social supports (23, 24), communication, and decision making (25, 26). While it appears necessary to develop and test dyadic interventions for persistent pain, it also appears evident that the interventions must be holistic, comprehensive, and multimodal to best meet the pain related needs of both individuals in the caregiving dyad (7). Additionally, many self-management skills, such as problem-solving and communication skills building, require two people to engage; thus, participating as a dyad may enhance skills and the relationship between persons with chronic conditions and their caregivers (27).

Management of persistent pain has historically included the use of pain medications, such as opioids, however, the current opioid epidemic has led to increased rates of addiction and overdose deaths (28). Even with opioid treatment, many patients continue to experience severe and disabling pain. Therefore, the development and testing of innovative non-pharmacological treatments to improve the management of persistent pain is needed.

Yoga is a non-pharmacological and holistic intervention which includes physical movements or postures (asanas), breath work (pranayama), and meditation (dhyana) and is more commonly being integrated into pain rehabilitation efforts. Evidence indicates that the connection of breath to physical movement allows for improved mind-body connection and awareness; this connection is considered beneficial and therapeutic (29–33). Results from our previous studies indicate that yoga is feasible and beneficial in multiple populations [e.g., stroke (34, 35), traumatic or acquired brain injury (36–43), Parkinson's Disease (44), older adults (45–47), cancer (48–50), and caregivers (51–53)], and is established as an intervention to improve pain-related outcomes for individuals with persistent pain (54–57). However, while beneficial, yoga does not include the development of skills considered necessary for optimization of pain or caregiving self-management, including: problem solving, action planning, effective communication, or coping skills (9, 58). The management of persistent pain requires day to day self-management (59); self-management interventions are developed so that participants can engage in their own self-care and treatment and have improved chronic pain outcomes (60, 61). While self-management education interventions are useful in managing pain, education alone is not enough as physical activity or movement is necessary for pain management.

To best address persistent pain among caregiving dyads, we developed and tested a new behavioral multi-modal intervention, the Merging Yoga and self-management to develop Skills (MY-Skills) intervention. The yoga intervention was based on prior work specific to persistent pain (57). The self-management skills education was developed specifically for this study. Intervention development included: focus groups with dyads with pain and clinicians with expertise in pain management to determine the needs of dyads with pain (62); a grounding in the Biopsychosocial Model (8); and development as a multimodal intervention, as MY-Skills merges yoga, a holistic physical activity, with self-management education. The purpose of this pilot study was to assess the feasibility, acceptability, and benefits of the MY-Skills intervention for caregiving dyads with chronic pain.

## 2 Materials and methods

### 2.1 Design and procedures

We completed a feasibility pilot study and assessed change in multiple outcomes after the in-person eight-week MY-Skills intervention. This was a randomized pilot study with a planned 1:1 allocation ratio. Assessments and the interventions took place on a university campus in Colorado, US.

Due to COVID-19, we adapted the in-person MY-Skills and control groups for online delivery. This paper includes results from both the in-person and online intervention. All procedures were approved by the university Institutional Review Board (#19-9095H); baseline and follow-up assessments were completed in person (on campus) or online by a trained, masked research assistant. All participants received educational handouts and supplies; the participants in MY-Skills received yoga supplies (yoga blocks, blankets) and participants in the control group received exercise supplies (hand weights, resistance bands).

### 2.2 Participants

Caregivers and care recipients with persistent pain were recruited through: medical providers at a local pain management clinic; study advertisements posted on local, university, state, and national organizations' websites; flyers sent to medical and allied health offices; and through social media groups targeting chronic pain and caregiving. To be eligible to participate in the in-person study, participants: lived within 50 mi of the university location, were a member of a caregiving dyad, were over the age of 18, communicated in English, reported chronic musculoskeletal pain for more than three months, had moderate or worse pain severity and interference (score of ≥5 on the Brief Pain Inventory**)** (63), were sedentary (<2, 30 min scheduled physical activities per week), scored ≥ 4 out of 6 on the short Mini-Mental State examination (64); were able to stand with or without an assistive device, and completed a 7-item Physical Activity Readiness Questionnaire + (PAR-Q+) (65). For the PAR-Q+, if participants responses were “yes” to any question, then physician clearance was required to participate in the yoga or physical activity portion of the study. For caregivers, additional inclusion criteria included providing care to the care receiver for at least six months. Criteria for the online study were maintained, except participants could live anywhere in the United States and were required to have access to a computer or electronic device with internet access and able to access online content. We planned for approximately 12 people in a group, as is the recommendation for pilot studies (66–68). We oversampled, and the original enrollment plan was to include 15 caregivers and 15 care receives in MY-Skills and in the control, thus 30 dyads (*N* = 60). However, due to moving the program online in the middle of the study, 17 individuals completed MY-Skills in person and 32 were online. A power analysis was not conducted as appropriate for a pilot study.

Additional exclusion criteria for all participants (for both in-person and online) included: having Alzheimer's disease or related dementia; having significant cardiovascular disease and/or myocardial infarction in the last three months; experiencing a stroke within the last six months; currently receiving cancer treatment (other than skin cancer); a life expectancy of less than one year; currently participating in rehabilitation for persistent pain for more than one time a week; currently in drug or alcohol treatment; participation in a yoga or exercise research study; or had completed self-management education within the last year. After participant screening was complete, all participants consented to the study and each participant received $50.00 as an incentive to complete an assessment before the intervention and $50.00 to complete an assessment after the intervention. Online participant incentives were in the form of $50.00 electronic gift cards.

### 2.3 Intervention and control groups

The MY-Skills and control group (Managing Your Pain through Learning And movement, MY-Plan) were designed as a group intervention, delivered in-person or online using a secure virtual platform, offered at the same frequency and duration (twice a week for 8 weeks, at two hours each session for approximately 32 h) to match time and contact with the interventionists. The in-person and online MY-Skills intervention and MY-Plan control group included 16 sessions, each with approximately 45–60 min of education and 60 min of yoga or physical activity.

#### 2.3.1 Randomization and masking

The biostatistician generated the random allocation sequence with a random number generator before baseline assessments. The assessor was masked and remained masked to group allocation throughout the study. Once both individuals in the dyad completed their baseline assessments, the dyad was randomized to MY-Skills or MY-Plan. The intent was to randomize at a 1:1 ratio to MY-Skills and MY-Plan. The project manager enrolled the participants, scheduled assessments, completed randomization, and notified the dyad about the group, meeting time, and location (on campus or virtual). Sealed envelopes were used to conceal study group allocation until the project manager notified the dyad about the study group, which was done after the completion of baseline assessments. The project manager and interventionists were not masked to group allocation. The groups were yoga and light exercise; thus participants knew if they were in yoga or exercise once the intervention began, however they were masked to knowing which was the intervention or the control group.

#### 2.3.2 MY-Skills intervention

The MY-Skills intervention was multi-modal, standardized, and progressive intervention that included education and yoga specific to meeting the needs of caregiving dyads experiencing persistent pain. The self-management education was developed specifically for this study; and was based on our discovery of what dyads with persistent pain and pain medical/allied health experts stated was needed in an educational intervention (62). To gather this information, we conducted focus groups with dyads with pain and clinicians with expertise in pain management (62) and completed a review of self-management and rehabilitation literature (61, 69, 70). Educational content for MY-Skills was developed to address pain, pain management, health, and the dyadic relationship (see Table 1 for the topics addressed in each session) (62) The group education was delivered by a trained research assistant who identified as a therapist (i.e., occupational therapist or marriage and family therapy). Self-management techniques included scripted lectures, discussions and brainstorming, and guided group activities to address common areas necessary in self-management education. For example, participants were guided by the trained interventionist in problem solving, weekly action planning, development of coping skills, and effective communication (58, 69). After the educational session, participants were given a 15-min break. During the break, participants were encouraged to review available resources, use the restroom, enjoy a light snack and water, while the interventionists transitioned and set-up for the yoga component.

**Table 1:** Summary of in-person merging yoga and self-management skills (MY-Skills) eight-week intervention.

Week	Session	Self-management of pain education topic	Yoga mantra	Yoga poses and breathwork
1	1.	Taking care of you	“I am here for my care”	All seated • Grounding (Centering)—connect to mantra • Intro to pranayama (breathwork) • Spinal twist and axial extension (spinal movement) • Head and neck and eye movements with prolonged holds • Receptive gesture, cactus arms (shoulder flexion, shoulder extension, shoulder rolls, and arm movements) • Mudras (finger movements) • Forward fold (forward flexion) • Half moon (lateral flexion) • Seated savasana (body scan with progressive relaxation)
	2.	Taking care of us and changing behaviors through action planning	“We are here for our care”	
2	3.	Pain 101 (Pain Education)	“I will help myself”	Last session plus: • Grounding (Centering)—connect to mantra and body scan • Pranayama (breathwork) • Ankle rotation • Move to standing (or remain seated if not able to safely stand) • Mountain pose (standing) • Receptive gesture, cactus arms (shoulder flexion, shoulder extension, shoulder rolls, and arm movements) • Half moon (lateral flexion) • Seated savasana (body scan with progressive relaxation)
	4.	Pain as a pair	“We will help each other”	
3	5.	Motivation and pain and action planning	“I am capable”	Last session plus: • Intro to diaphragmatic breathing and extended exhalation • Move to standing (or remain seated if not able to safely stand) • Crescent moon (standing supported lunges) • Locust pose in standing (hip extension while standing) • Seated savasana (body scan with progressive relaxation)
	6.	Body mechanics and action planning	“We are capable”	
4	7.	Stress management	“Breathe in quiet, breathe out calm”	Last session plus: • Grounding (Centering)—connect to mantra and check in with body and emotions • Alternate nostril breathing • Intro to partner poses while seated– shoulder abduction and touching partner's hand • Standing postures • Move to floor or chair • Intro to coming down to floor • Savasana on back or in chair
	8.	Dealing with difficult emotions and action planning	“Breathe in quiet, breathe out calm”	
5	9.	Communication as a pair	“I am strong. Together we are stronger”	Last session plus: • Partner pose—shoulder abduction, touching partner's hand, adding silently saying the mantra (“I am strong. Together we are stronger”) • Standing postures • Move to floor or chair • Seated or supine pigeon (figure four) • Cactus arms • Savasana on back or in chair
	10.	Communication with your medical team medication management and action planning	“I am strong. Together we are stronger”	
6	11.	Fatigue and pain	“I will work with my energy””	Last session plus: • Grounding (Centering) connect to mantra and check in with body and energy levels • Partner pose—(silently repeat mantra and visualize giving and receiving support with hand touching) • Standing postures • Move to floor • Bridge pose (supine hip extension) • Wind removing pose (hip and knee flexion to one leg at a time) • Savasana on back on in the chair
	12.	Activity modification and action planning	“We will work with our energy”	
7	13.	Healthy eating and pain	“I choose health”	Last session plus: • Partner pose—(mirroring technique seated across from each other and gazing into each other's eyes while touching hands and silently repeating mantra) • Standing postures • Crescent moon (supported lunge) to warrior I • Savasana on back or in chair
	14.	Yoga as physical activity Yoga and pain and action planning	“We choose health”	
8	15.	Applying skills and forming habits and routines	“I can help myself”	Last session plus Q/A and participant feedback
	16.	Long-term action planning and wrap up	“We can help each other”	

The yoga intervention was based on prior work specific to persistent pain (57) and was modified for dyads with chronic pain to ensure success throughout the yoga component (71). We included Hatha yoga which is considered to be a gentle form of yoga where movement is connected with breath and the yoga pose may be held for multiple breaths. This allows the individual to stay in a pose to enhance strength and flexibility and “settle” into a pose, which is thought to improve body awareness (72). The standardized yoga intervention was delivered by a yoga therapist and tailored to individual needs to allow for personal success and improvement. For example, not everyone completed all poses or moved to the floor to complete yoga, as their pain varied. Yoga was planned to be progressively challenging over the eight weeks and included seated, standing, and floor postures. All yoga sessions included breathwork, yoga poses, connection of movement with breath, mantras, and meditation. During week four, we introduced “partner poses” where both members of the dyad connected in some way (e.g., touching palms while standing, making eye contact during poses). To merge the two interventions (education and yoga), each educational session included information and a weekly mantra to connect the education topic to the use of yoga for pain management. The yoga interventionist wove the mantra into the yoga session, delivered after the educational component. See Table 1 for additional details.

#### 2.3.3 MY-Plan control group

The MY-Plan control group was developed to match the metabolic expenditure of yoga and included health and wellness education and light exercise, not specific to pain management (73). MY-Plan was a standardized group program delivered by trained exercise physiologists. The program sessions did not include key components of MY-Skills or Chronic Disease Self-management (CDSM) and the interventionists were trained when asked questions by participants to avoid topics similar to MY-Skills (i.e., action planning, coping, etc.). The educational topics included general health promotion such as heart health, cancer prevention, and healthy eating habits. Physical activity included a warmup and cool down, as well as walking, balance, using resistance bands, weightbearing activities, and core work, but did not include yoga or breath work.

#### 2.3.4 Modifications for online delivery of My-Skills and My-Plan

Due to COVID-19, all in-person sessions halted in March 2020 and modifications were made to move MY-Skills and My-Plan to online. For example, prior to the delivery of the online intervention and control group, we adapted the flip charts and discussion boards used during the in-person MY-Skills and MY-Plan and created PowerPoint slides for each session. In addition, content that was scripted for interventionists was also put on visual slides to guide and engage participants during the online groups. All baseline and follow-up assessments were completed online, and all participants received educational handouts and yoga or exercise supplies in the mail after baseline assessments were complete.

Prior to the first online MY-Skills or MY-Plan session, the yoga or physical activity interventionists met with each participant to go over their computer set-up to ensure proper positioning of their chair and the camera for the yoga or physical activity component. Participant safety was of utmost importance, and in addition to proper set-up, the interventionists collected telephone information of each person and an emergency contact in case anything happened during the intervention. Further, a yoga or physical activity assistant was on the video call to monitor participants and their movements. If any concerns occurred, the assistant would immediately notify the interventionist delivering the yoga or physical activity. Finally, moving from in-person to an online intervention meant needing to further modify the yoga portion of MY-Skills. Although yoga was progressively challenging over the eight weeks and included seated and standing postures, the yoga interventionist no longer introduced floor postures due to safety concerns of being online.

### 2.4 Measures

#### 2.4.1 Feasibility

Feasibility was the primary aim of the study. Feasibility data were collected throughout the study. We assessed the following aspects of feasibility: recruitment, consent, attendance, attrition, ability to complete the intervention and assessments, safety, and accessibility and acceptability/satisfaction. Prior to the study, benchmark criteria were determined for each aspect of feasibility and acceptability (Table 2).

**Table 2:** Feasibility and acceptability benchmarks and study results for in-person and online pilot studies.

Feasibility or acceptability construct	Benchmark set a prior	Results from the in-person pilot	Results from the online pilot
Recruitment	Expect 30% of screened dyads will be eligible to participate	18%: Of the 200 screened individuals, 36 (18 dyads) were eligible to participate	50%: Of the 80 screened individuals, 40 (20 dyads) were eligible to participate
Consent	Expect 30% of eligible dyads will consent	36%: Of the 18 dyads eligible, 7 dyads (13 individuals, one dropped out) consented	80%: Of the 20 dyads eligible, 16 dyads (31 individuals, one dropped out) consented
Attendance (and reasons recorded)	Expect ≥50% of sessions	65% of sessions attended, reasons for absences recorded	85% of sessions attended, reasons for absences recorded
Attrition (and reasons recorded)	Expect ≤20% attrition	31%: 4 individuals	3%: 1 individual
Assessments and intervention completed	90% of enrolled participants will complete the intervention and ≤1.5 hours of assessment	100%: all 9 individuals who completed the intervention were assessed (M = 51 minutes, no assessments exceeded 1.5 h) and 70% completed the intervention	100%: all 30 individuals who completed the intervention were assessed (*M* = 1 hour and 23 minutes, 11 assessments exceeded 1.5 hrs) and 97% completed the intervention
Safety tracked via adverse events (AE)	Expect ≤3 (10%) participants to sustain a serious adverse event	0 AE related to the intervention 17%: 3 participants experienced AE unrelated to the study	0 AE related to the intervention 6%: 2 participants experienced AE unrelated to the study
Acceptability and satisfaction	Expect 90% of participants to rate MY-Skills between 4-7 (on a 7-point Likert scale, 1 = not satisfied, 7 = very satisfied), indicating acceptability and satisfaction	100%: 100% of participants who completed the post-assessment rated their satisfaction (on average) of 5 or greater	100%: 100% of participants who completed post-assessment rated their satisfaction (on average) of 4 or higher

#### 2.4.2 Participant demographics

Demographic characteristic data and outcome measures were collected by a masked and trained research assistant. Demographic characteristic data included: age, gender, marital status, race, ethnicity, and education level. Information about pain was also collected (i.e., time with pain, use of pain medications, etc.).

#### 2.4.3 Outcome measures

All outcome measures were assessed before and after completion of the eight-week intervention or control group. The assessor was masked to group allocation. Targeting outcomes based on the Biospychosocial Model, outcomes of interest included physical health (including pain interference and severity), mental health, health related quality of life, and occupational performance and satisfaction. Outcomes measures were collected primarily to determine the feasibility of outcome data collection rather than to test efficacy as the study was not powered to do so.

##### 2.4.3.1 Pain

Brief Pain Inventory (BPI) was used to measure participants' pain interference on daily life and activities and pain severity (63). The BPI is a valid and reliable measure and includes 11 items (total score) with seven items used to assess pain interference (or pain related disability) and four items to assess pain severity. The BPI includes means scores for the total score and two subscales, with lower scores indicating less pain interference and severity (63, 74).

##### 2.4.3.2 Health-related quality of life

The SF-12v2 is a measure of health-related quality of life (QoL) and includes 12 items to measure eight health domains (75) where higher scores indicate higher health-related QoL. The SF-12v2 is valid and reliable and has been used in studies of people with chronic pain. Two scores are calculated, one for physical health and well-being and one for mental health and well-being.

##### 2.4.3.3 Physical and mental health

The NIH PROMIS-29 Profile (v2.0) was used to assess multiple important health factors, including: pain intensity; pain related-disability; anxiety; depression; fatigue; physical functioning; sleep disturbance; and ability to participate in social roles and activities (76). The assessment includes 29 items, divided into scales for each health factor. The scoring of each scale varies, with some high scores representing improvement (such as sleep quality) and other high scores representing a decrease (such as pain intensity). The total scale score is calculated by reverse-scoring appropriate items to align indicators of improvement. Once this process is complete, lower scores indicate improvement (such as decreases in depressive symptoms).

##### 2.4.3.4 Occupational performance and satisfaction

The Canadian Occupational Performance Measure (COPM) was used to assess performance and satisfaction with performance on five activities (occupations) (77). The COPM is commonly used in occupational therapy and was completed using a semi-structured guide to help the participant identify activities that were important yet challenging. The participant then ranked the five most important activities and rated their ability to perform the activity and their satisfaction with their performance of the activity. A two-point change indicates clinical significance. The COPM has been used with chronic pain and in yoga and pain studies; higher scores indicate greater occupational performance as well as greater satisfaction with their perceived performance (78, 79).

### 2.5 Data analysis

Data entry occurred in RedCap (for in-person sessions) and Qualtrics (for online sessions) and analyses were completed using Statistical Package for the Social Sciences 26 (IBM Corp., 2019). The biostatistician guided the use of descriptive statistics (e.g., means and standard deviations) to describe demographic characteristics, pain, and feasibility. This study was funded by the National Institute of Health National Center for Complementary and Integrative Health (NCCIH). NCCIH guidelines for pilot studies indicate that no inferential statistics would be proposed or used (80). Thus, outcome measure data are described, but not analyzed, for caregivers and care receivers for MY-Skills and MY-Plan for in-person and online.

## 3 Results

### 3.1 Participants

Six caregiving dyads were randomized into the in-person MY-Skills intervention for a total of thirteen individuals (caregivers *n* = 7, care receivers *n* = 6). Caregiver and care receiver numbers were not equal as one dyad terminated their relationship and the care receiver identified a new caregiver. The newly added caregiver baseline data are included; however, the caregiver did not complete post-intervention assessments as the participant did not complete the full intervention. The average age of the caregivers in the in-person MY-Skills was 48.6 ± 17.88, 71% were female participants, and 71% completed at least some college education. The care receivers had an average age of 52.17 ± 11.46 years, were 83% female participants, and 66% completed at least some college education. Four individuals were randomized to the in-person MY-Plan control group. Most participants (58%) in the in-person group had pain for more than 10 years.

The online MY-Skills group included 18 participants (nine dyads) at baseline and the average age of caregivers was 60.33 ± 20.72 and 65 ± 18.97 for the care receivers. Fourteen individuals (seven dyads) were randomized to the online MY-Plan. The majority of online participants were female and college-educated. Half (50%) of online participants had pain for over 10 years. See Table 3 for all demographic information. Of note, in-person and online MY-Skills and MY-Plan groups were not equal in number as full recruitment was not reached. Had the planned sample been attained, there would have been an equal number of participants randomized to each group. Recruitment stopped once the pre-established number of cohorts were filled for both MY-Skills and MY-Plan.

**Table 3:** Demographic characteristic (e.g., age, gender) descriptive statistics (e.g., mean (SD) or *n* (%) for caregivers and care receivers.

	In-person caregivers		In-person care receivers		Online caregivers		Online care receivers	
	MY-Skills	Control	MY-Skills	Control	MY-Skills	Control	MY-Skills	Control
	*n* = 7	*n* = 2	*n* = 6	*n* = 2	*n* = 9	*n* = 7	*n* = 9	*n* = 7
Age, mean (SD)	48.57 (17.88)	45.00 (21.21)	52.17 (11.46)	59.50 (36.06)	60.33 (20.72)	37.57 (12.73)	65.00 (18.97)	39.86 (21.04)
Age range	25–72 years	30–60 years	30–60 years	34–85 years	31–90 years	24–57 years	27–86 years	24–80 years
Gender, *n* (%)								
Male	2 (29%)	0	1 (17%)	1 (50%)	3 (33.3%)	6 (86%)	1 (11%)	0
Female	5 (71%)	2 (100%)	5 (83%)	1 (50%)	6 (66.7%)	1 (14%)	8 (89%)	6 (86%)
Relationship status, *n* (%)								
Partnered	3 (43%)	1 (50%)	2 (33%)	1 (50%)	4 (44%)	4 (57%)	5 (56%)	3 (43%)
Not partnered	4 (57%)	1 (50%)	4 (67%)	1 (50%)	5 (56%)	3 (43%)	4 (44%)	4 (57%)
Race, *n* (%)								
Black	0	0	1 (11%)	0	1 (11%)	0	1 (11%)	0
White	7 (100%)	1 (50%)	5 (83%)	2 (100%)	7 (78%)	6 (86%)	8	7 (100%)
Asian American	0	1 (50%)	0	0	0	0	0	0
Hispanic/Latino	0	0	0	1 (14.3%)	0	1 (14%)	0	0
Other	0	0	1 (11%)	0	1 (11%)	0	0	0
Ethnicity, *n* (%)								
Hispanic/Latino	0	0	0	0	1 (11%)	1 (14%)	1 (11%)	0
Not Hispanic/Latino	5 (71%)	2 (100%)	6 (100%)	2 (100%)	7 (78%)	4 (57%)	7 (78%)	5 (71%)
Other or no answer	1 (14%)	0	0	0	1 (11%)	2 (29%)	1 (11%)	2 (29%)
Education, *n* (%)								
Some high school	0	0	1 (17%)	0	0	0	0	0
High school graduate	2 (29%)	0	1 (17%)	0	0	0	1 (11%)	1 (14%)
Some college	5 (71%)	1 (50%)	4 (67%)	0	3 (33%)	1 (14%)	4 (44%)	3 (43%)
College graduate	0	1 (50%)	0	0	6 (67%)	6 (86%)	4 (44%)	3 (43%)
How long ago did you start having chronic pain?								
<10 years	3 (43%)	1 (50%)	1 (17%)	2(100%)	5 (55.6%)	3 (43%)	3 (33%)	4 (57%)
≥10 years	4 (57%)	1 (50%)	5 (83%)	0	4 (44.4%)	4 (57%)	6 (67%)	2 (29%)
Do you take medication for chronic pain? (yes)	5 (71%)	1 (50%)	5 (83%)	1 (50%)	7 (77.8%)	3 (43%)	8 (89%)	4 (57%)
Are you taking opioids? (yes)	4 (57%)	0	5 (83%)	1 (50%)	5 (55.6%)	0	4 (44.4%)	2 (28.6%)

Frequencies may not sum to 100% due to unreported/missing data (e.g., due to participant attrition).

Caregiver and care receiver numbers were not equal as one dyad terminated their relationship and the care receiver identified a new caregiver. The newly added caregiver baseline data are included; however, the caregiver did not complete post-intervention assessments as the participant did not complete the full intervention.

### 3.2 Feasibility

**Figure 1:**
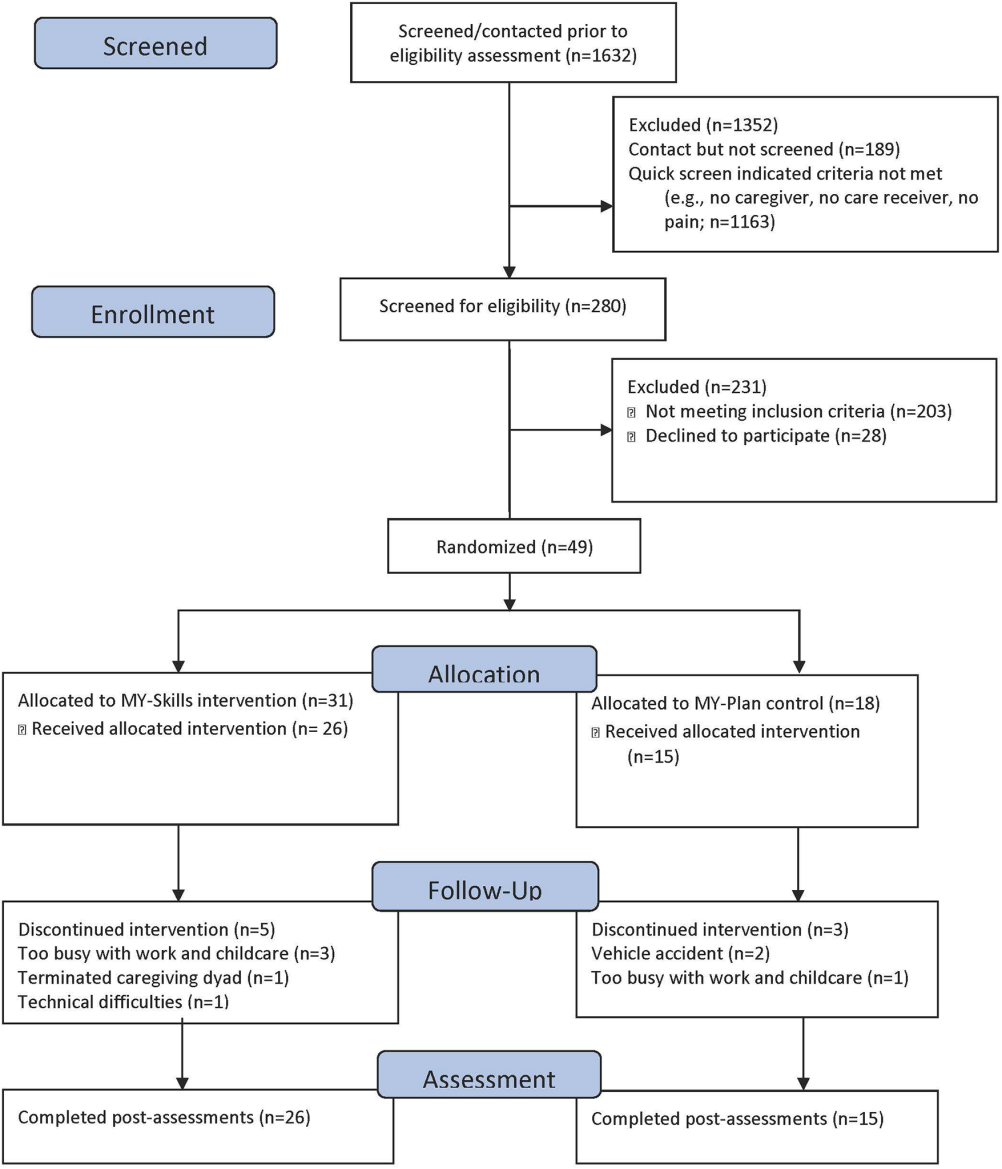
Consort Diagram for MY-Skills.

Feasibility was the primary outcome of the pilot study. Benchmarks related to recruitment and attrition were not met for the in-person pilot study. For example, only 18% of the 200 fully screened individuals met criteria to be eligible to participate in the study (see Figure 1 for the Consort Diagram and Table 2). Primary reasons for ineligibility included: not being part of a caregiving dyad (i.e., not having a caregiver or someone that helps them), both members of the dyad not having moderate pain or a sedentary lifestyle, and inability to commit to the twice per week, 8-week intervention. Four individuals dropped out of the study; however, it is noted that one dyad was in a vehicular accident and sustained injuries that caused them both to stop the study and one individual was the caregiver in the dyad who terminated the relationship. Other benchmark criteria, such as attendance, completion of the assessments, completion of the intervention, and safety, were exceeded for the in-person MY-Skills intervention (see Table 2). Related to acceptability and satisfaction, 100% of the participants who completed the post-intervention assessment indicated a “5” or higher (scale of 1–7, 7 = very satisfied) on a satisfaction scale asking about the MY-Skills intervention. There were no adverse events related to the intervention.

The online MY-Skills intervention was found to be feasible, acceptable, and safe (see Table 2). All benchmarks were met or were exceeded for the online study. Online recruitment was feasible with 50% of fully screened individuals being eligible for the study, and 80% of eligible dyads consented to be in the study. As in the in-person study, there were not any adverse events related to the study, there were no reported unintended effects, and 100% of participants who completed the final assessments rated their satisfaction as a 4 or higher (scale of 1–7, 7 = very satisfied). For both the in person and online yoga and control groups, a fidelity check-off list was developed and completed by a research assistant. Weekly team meetings included review of the fidelity check-off lists and the team worked with the interventionists to increase fidelity as needed.

Of note, over 1,000 individuals had contact with the study team but were not fully screened for in-person or online eligibility. The reasons participants were not fully screened included that: a) immediately following the short study description, the individual disclosed to the screener that they were not part of a dyad; b) the caregiver did not report pain; or c) the individual reported physical activity (not meeting the sedentary criteria).

### 3.3 Preliminary intervention benefits

**Figure 2:**
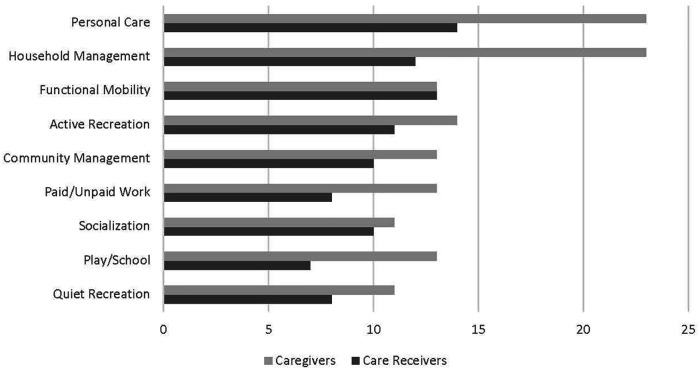
The number of times that challenging areas of concern, according to the Canadian occupational performance measure, were reported by caregivers and care receivers for all MY-Skills and MY-Control particpants (*N* = 49).

Related to the included outcome measures, individuals in four dyads (*n* = 9) completed the in-person MY-Skills intervention and post-intervention data collection (Table 4). Of dyads who participated in the MY-Skills intervention, care receivers had a decrease in pain severity and pain interference, and overall better scores on outcome measures than the caregivers (see Table 4). In general, there were improvements in physical and mental health and well-being (SF-12 and PROMIS-29 scores). Both the caregivers and care receivers demonstrated improved occupational performance and satisfaction, with over 50% relative improvement in COPM satisfaction scores (Time 1—Time 2 divided by Time 1, multiplied by 100). In-person MY-Skills caregivers and care receivers demonstrated a two-point change (clinical significance) in COPM satisfaction. Of note, the COPM activities identified by caregivers and care receives seem to differ (see Figure 2). Two dyads completed the MY-Plan control, however, only one dyad completed all data collection post-intervention. The participants in the MY-Plan control group demonstrated improved pain related outcomes.

**Table 4:** Descriptive statistics [e.g., mean (SD)] for outcome measures for In-person MY-Skills and MY-Plan.

	In-person MY-Skills intervention				In-person MY-Plan control			
	Caregiver		Care receiver		Caregiver		Care receiver	
	Baseline (*n* = 7)	Post-intervention (*n* = 4)	Baseline (*n* = 6)	Post-intervention (*n* = 5)	Baseline (*n* = 2)	Post-intervention (*n* = 1 or 2)	Baseline (*n* = 2)	Post-intervention (*n* = 1 or 2)
BPI pain severity	6.71 (1.46)	7.00 (0.84)	7.42 (0.79)	6.95 (1.16)	6.63 (1.24)	5.88 (0.18)	6.63 (1.94)	4.50 (2.83)
BPI pain interference	7.20 (0.81)	7.86 (1.38)	7.00 (1.43)	5.11 (2.23)	4.64 (2.53)	4.71 (1.82)	7.14 (1.62)	6.21 (4.34)
SF-12v2 physical health and well-being	36.61 (7.89)	38.93 (3.84)	32.19 (6.98)	40.44 (3.13)	42.54 (10.66)	41.02 (17.47)	32.84 (0.17)	40.20
SF-12v2 mental health and well-being	41.43 (4.75)	40.47 (9.83)	40.35 (9.25)	43.46 (3.00)	32.52 (2.87)	42.07 (11.08)	38.28 (7.94)	33.22
PROMIS-29^	100.29 (7.43)	95.00 (12.06)	100.20 (15.24)	92.00 (21.79)	90.00 (9.90)	86.50 (24.75)	95.00	107.00
COPM performance	5.09 (1.01)	6.85 (0.41)	5.32 (1.14)	6.93 (0.90)	5.10 (0.42)	6.00	5.20	4.60
COPM satisfaction	4.11 (2.80)	6.25 (0.91)	3.84 (1.45)	5.87 (0.70)	4.00 (0.57)	5.40	5.20	3.60

BPI, brief pain inventory; SF, short form health survey; PROMIS-29, PROMIS profile physical and mental health summary score, ^lower scores indicate improvement; COMP, Canadian occupational performance measure.

Control group data are incomplete due to missing data (*n* = 1) for some outcome measures; there is no standard deviation.

Nine dyads (*n* = 18) began the online MY-Skills intervention and one care receiver dropped out due to a work schedule conflict (Table 5). Regarding the online intervention, there are improvements noted for physical and mental health and well-being and PROMIS-29 scores. The MY-Plan online control group also showed decreased pain severity and pain interference.

**Table 5:** Descriptive statistics [e.g., mean (SD)] for outcome measures for online MY-Skills and MY-Plan.

	Online MY-Skills intervention				Online MY-Plan control			
	Caregiver		Care receiver		Caregiver		Care receiver	
	Baseline (*n* = 9)	Post-intervention (*n* = 9)	Baseline (*n* = 9)	Post-intervention (*n* = 8)	Baseline (*n* = 7)	Post-intervention (*n* = 6)	Baseline (*n* = 7)	Post-intervention (*n* = 7)
BPI pain severity	6.00 (1.48)	6.03 (1.11)	6.78 (1.63)	6.97 (1.69)	5.50 (0.94)	4.54 (0.89)	6.43 (1.27)	5.75 (2.05)
BPI pain interference	5.65 (2.49)	6.08 (2.03)	6.84 (1.65)	6.36 (1.95)	5.86 (1.42)	4.00 (2.31)	7.49 (2.05)	5.53 (2.72)
SF-12v2 physical health and well-being	34.96 (5.67)	39.42 (5.42)	37.04 (4.25)	35.74 (4.84)	43.50 (3.15)	42.37 (2.81)	40.23 (7.02)	39.76 (6.11)
SF-12v2 mental health and well-being	44.66 (3.24)	44.93 (5.05)	44.06 (2.25)	45.85 (4.00)	44.00 (5.55)	42.47 (4.13)	42.25 (4.46)	44.19 (3.98)
PROMIS-29^	78.56 (12.05)	72.22 (11.55)	80.22 (16.08)	72.88 (12.83)	69.17 (12.47)	63.50 (13.53)	88.57 (3.95)	82.86 (13.17)
COPM performance	6.93 (1.93)	6.92 (2.17)	7.07 (2.10)	6.50 (2.26)	6.00 (3.01)	7.57 (0.61)	5.40 (1.53)	5.51 (2.14)
COPM satisfaction	5.93 (2.92)	5.84 (2.33)	6.51 (2.64)	6.15 (2.34)	5.26 (3.50)	6.77 (1.74)	3.77 (1.36)	4.40 (2.05)

BPI, brief pain inventory; SF, short form health survey; PROMIS-29,PROMIS profile physical and mental health summary score, ^lower scores indicate improvement; COMP,Canadian occupational performance measure.

## 4 Discussion

The purpose of this study was to examine the feasibility, acceptability, and preliminary benefits of the eight-week MY-Skills intervention for caregiving dyads. After data from baseline and post-test assessments were examined, we found that all feasibility benchmark criteria were met or exceeded for the online MY-Skills study and that the in-person study did not meet recruitment or attrition criteria, but met or exceeded all other benchmarks. Both the online and in-person interventions appear acceptable and descriptive trends indicate that participants may have benefited from the intervention. Recruitment could be enhanced by modifying the inclusion and exclusion criteria of participants. For example, a future study should not require participants to be sedentary or for caregivers to have pain. When screening possible participants, people with persistent pain often stated their medical providers encouraged them to engage in physical activity and to exercise.

Similar to previous research supporting dyadic interventions with caregiving dyads experiencing persistent pain (21, 22), MY-Skills in-person and online interventions were acceptable and met most feasibility benchmarks. Recruitment of participants into the study had better results for MY-Skills online (50% of those screened were eligible to participate) than for the in-person intervention (18% were eligible). This is interesting given the only eligibility criteria that changed were expanding to outside of the community and the need for a computer with a camera and internet access. The additional recruitment strategies (i.e., nationwide social media websites) and exclusion of living in the community close to the intervention location may have increased eligible participants for the online MY-Skills version. It is possible that the online delivery of MY-Skills removed barriers to in person interventions, such as driving, parking, or fear of movement or of leaving the home. The online delivery may have allowed individuals with persistent pain to complete an intervention they would otherwise not be able to attend. These study results echo a recent systematic review related to the acceptability and feasibility of hatha yoga delivered online (72). Brosnan and colleagues reviewed ten articles related to the delivery of online hatha yoga and determined that is appears online yoga is feasible and can potentially improve outcomes and symptoms of multiple diagnoses. As noted, Brosnan et al. (72) indicated online yoga is feasible, acceptable, and beneficial for multiple ages and diagnose, however it appears online yoga, plus education for dyads, for persistent pain has yet to be explored.

Our attendance and attrition was similar to previous studies with small sample sizes and eligibility rates enrolled into in-person dyadic interventions (22, 81). Attendance exceeded our *a priori* rate of 50%, with 65% of sessions attended for in-person and 85% of sessions attended for online. Differences in attrition were 31% for in-person and only 3% for online MY-Skills; perhaps indicating that in-person interventions are dependent on both individuals in the dyad being able and interested in participating or that attending an online intervention is less burdensome on participants' total time (i.e., when considering travel to the intervention site and time of day for intervention delivery). However, attrition rates could be impacted by timing and COVID-19, for example, vaccines were not yet available and participants were potentially isolated. Participating in the study was a way to connect with others. Likely the attendance and attrition rates for the online intervention are somewhat skewed.

Of those participants who remained in the study, 100% of participants who completed baseline assessments also completed the post-assessment. This completion rate could be associated with two reasons: (a) participant incentives directly connected to assessments, and (b) people had time and interest in engaging with others due to limited social interactions related to COVID-19. Most in-person assessments were completed in less than 60-min; however, 11 assessments conducted online exceeded 90 min. This is in opposition to research suggesting the telehealth visits online are shorter than those in-person (82). It could be that the communication during the in-person assessments was easier than the online assessments, as communication between the assessor and the participant was more burdensome for online participants. Alternatively, participants might have been more interested in talking with the assessor during the online MY-Skills assessment as the pandemic might have contributed to loneliness and isolation and therefore the time it takes to assess participants should be further explored. Finally, the satisfaction surveys that we administered during the in-person and online MY-Skills showed that 100% of participants were satisfied with the intervention, which is common among both yoga and self-management studies, and is shown to be associated with improved intervention outcomes (83).

As this was a pilot study, inferential statistics were not employed, however our pilot study results indicate that MY-Skills may lead to benefits in physical, mental, and quality of life domains, but further efficacy testing is needed. Mean scores for pain severity and interference did not decrease as expected. Changes in outcome measures trended in the positive direction for care receivers' and not for caregivers. This is not surprising given caregivers tend to do worse over time and may experience greater stressors and strains in their daily lives (12) and may experience increases in fatigue. Yet, yoga is known to decrease stress and fatigue (84) and further research on the impact MY-Skills has on these outcomes should be explored. Pain related benefits may not have been identified because participants might have paid more attention to their persistent pain and health behaviors during, and as a result of, the MY-Skills classes, as they identified more areas for improvement. Participants may not have been as aware of major issues identified in the literature [i.e., mental health and caregiver/receiver strain (15)] before the class began and, as a result, scored themselves higher on the scales after the intervention. Thus, increased awareness over the course of the intervention might have led them to score themselves similarly or lower during post-assessment. We did identify that participants in the in-person MY-Skills improved their occupational performance and satisfaction; yet, because of the nature of this semi-structured interview measure, results from online MY-Skills may not be a reliable indicator of self-perceptions of performance in self-care, leisure, and productivity. Large scale efficacy trial testing is needed to determine the intervention effect on biopsychosocial outcomes.

Noted differences between in-person and online groups may have an impact on outcome measure scores. For example, the “group interaction” was inherently different between in-person and online groups; the online participants did not ask questions of the interventionists and did not seem to connect as the in-person group members connected. Additionally, it is common to include local resources during self-management education, however the online international format meant the resources were not tailored locally. Lastly, as documented by other researchers (85), we experienced internet connectivity problems for both participants and interventionists, this likely means the intervention was not delivered as planned and it is important to adequately prepare for connectivity issues and monitor online fidelity (86).

A strength of this study includes the establishment of feasibility and acceptability of both the in-person and online MY-Skills 8-week intervention. Another strength of this study was our attention to the development of the control group to carefully match the level of activity with the level of activity in the intervention group, contributing to the established gap of knowledge on control groups in yoga literature (87). Additionally, during the development of the MY-Plan educational component, we made certain not to address the elements commonly found in chronic disease self-management education (61). Based on the study results, people with persistent pain need education, activity, and a shared experience with their caregiver and/or care receiver; as a result, both the intervention and control might have helped participants.

### 4.1 Limitations

Limitations of this pilot study include a small sample size not allowing for generalizability or to use as preliminary establishment of efficacy. The rapid approach to moving the multi-modal intervention online and unavoidable challenges of a global pandemic may limit the feasibility and acceptability of MY-Skills in the future. Thus, at this time, there is remaining uncertainty about feasibility.

## 5 Conclusion

In conclusion, MY-Skills (both in-person and online) may be a feasible and acceptable intervention for dyads experiencing pain and could enhance pain rehabilitation efforts. However, future trials will require adjusted recruitment criteria so that successful and timely recruitment of dyads is achievable. Future research testing of the MY-Skills intervention should be considered with larger sample sizes to further establish efficacy of health-related outcomes for the caregiving dyad experiencing persistent pain. Not only will this next step further elevate the support for MY-Skills, but it will also allow for refinement of content delivered during the self-management educational sessions. Additionally, in the future, MY-Skills could be considered for other populations with different types of pain or other disability. With established feasibility, future researchers should continue to address the needs of dyads through multi-modal interventions combining self-management education and physical activity, including yoga.

## Data Availability

The raw data supporting the conclusions of this article will be made available by the authors, without undue reservation.

## References

[1] Goldberg DS McGee SJ. Pain as a global public health priority. BMC Public Health. (2011) 11(1):770. 10.1186/1471-2458-11-770 21978149 PMC3201926

[2] Pitcher MH Von Korff M Bushnell MC Porter L. Prevalence and profile of high-impact chronic pain in the United States. J Pain. (2019) 20(2):146–60. 10.1016/j.jpain.2018.07.006 30096445 PMC8822465

[3] Persson E Lexell J Rivano-Fischer M Eklund M. Occupational performance and factors associated with outcomes in patients participating in a musculoskeletal pain rehabilitation programme. J Rehabil Med. (2014) 46(6):546–52. 10.2340/16501977-1810 24819126

[4] Persson E Lexell J Rivano-Fischer M Eklund M. Everyday occupational problems perceived by participants in a pain rehabilitation programme. Scand J Occup Ther. (2013) 20(4):306–14. 10.3109/11038128.2013.793739 23621672

[5] Persson E Rivano-Fischer M Eklund M. Evaluation of changes in occupational performance among patients in a pain management program. J Rehabil Med. (2004) 36(2):85–91. 10.1080/16501970310019142 15180223

[6] Fisher G Emerson L Firpo C Ptak J Wonn J Bartolacci G. Chronic pain and occupation: an exploration of the lived experience. AJOT. (2007) 61(3):290–302. 10.5014/ajot.61.3.290 17569386

[7] NIH. National pain strategy: A comprehensive population health-level strategy for pain. (2016). Available online at: https://iprcc.nih.gov/docs/HHSNational_Pain_Strategy.pdf (Accessed June 30, 2022).

[8] Gatchel RJ Peng YB Peters ML Fuchs PN Turk DC. The biopsychosocial approach to chronic pain: scientific advances and future directions. Psychol Bull. (2007) 133(4):581–624. 10.1037/0033-2909.133.4.581 17592957

[9] Bair MJ Ang D Wu J Outcalt SD Sargent C Kempf C Evaluation of stepped care for chronic pain (ESCAPE) in veterans of the Iraq and Afghanistan conflicts: a randomized clinical trial. JAMA Intern Med. (2015) 175(5):682–9. 10.1001/jamainternmed.2015.97 25751701

[10] McCracken LM Eccleston C. Coping or acceptance: what to do about chronic pain? Pain. (2003) 105(1-2):197–204. 10.1016/S0304-3959(03)00202-1 14499436

[11] Jones SL Hadjistavropoulos HD Janzen JA Hadjistavropoulos T. The relation of pain and caregiver burden in informal older adult caregivers. Pain Medicine. (2011) 12(1):51–8. 10.1111/j.1526-4637.2010.01018.x 21143758

[12] Schulz R Beach SR. Caregiving as a risk factor for mortality: the caregiver health effects study. J Am Med Assoc. (1999) 282(23):2215–9. 10.1001/jama.282.23.2215 10605972

[13] Pinquart M Sörensen S. Differences between caregivers and noncaregivers in psychological health and physical health: a meta-analysis. Psychol Aging. (2003) 18(2):250–67. 10.1037/0882-7974.18.2.250 12825775

[14] Zarit SH. Family Caregiving: Agenda for the Future. Cantor MH, editor. San Francisco, CA: American Society on Aging (1994).

[15] Lyons KS Zarit SH Sayer AG Whitlatch CJ. Caregiving as a dyadic process: perspectives from caregiver and receiver. J Gerontol B Psychol Sci Soc Sci. (2002) 57(3):195–204. 10.1093/geronb/57.3.P195 11983730

[16] Savini S Buck HG Dickson VV Simeone S Pucciarelli G Fida R Quality of life in stroke survivor–caregiver dyads: a new conceptual framework and longitudinal study protocol. J Adv Nurs. (2015) 71(3):676–87. 10.1111/jan.12524 25186274

[17] Whitlatch CJ Judge K Zarit SH Femia E. Dyadic intervention for family caregivers and care receivers in early-stage dementia. Gerontologist. (2006) 46(5):688–94. 10.1093/geront/46.5.688 17050761

[18] Badr H Smith CB Goldstein NE Gomez JE Redd WH. Dyadic psychosocial intervention for advanced lung cancer patients and their family caregivers: results of a randomized pilot trial. Cancer. (2015) 121(1):150–8. 10.1002/cncr.29009 25209975 PMC4270818

[19] Milbury K Chaoul A Engle R Liao Z Yang C Carmack C Couple-based Tibetan yoga program for lung cancer patients and their caregivers. Psycho-Oncology. (2015) 24(1):117–20. 10.1002/pon.3588 24890852 PMC4437691

[20] Milbury K Mallaiah S Lopez G Liao Z Yang C Carmack C Vivekananda yoga program for patients with advanced lung cancer and their family caregivers. Integr Cancer Ther. (2015) 14(5):446–51. 10.1177/1534735415583554 25917816 PMC4537807

[21] Keefe FJ Blumenthal J Baucom D Affleck G Waugh R Caldwell DS Effects of spouse-assisted coping skills training and exercise training in patients with osteoarthritic knee pain: a randomized controlled study. Pain. (2004) 110(3):539–49. 10.1016/j.pain.2004.03.022 15288394

[22] Abbasi M Dehghani M Keefe F Jafari H Behtash H Shams J. Spouse-assisted training in pain coping skills and the outcome of multidisciplinary pain management for chronic low back pain treatment: a 1-year randomized controlled trial. Eur J Pain. (2012) 16(7):1033–43. 10.1002/j.1532-2149.2011.00097.x 22337646

[23] Boise L Congleton L Shannon K. Empowering family caregivers: the powerful tools for caregiving program. Educ Gerontol. (2005) 31(7):573–86. 10.1080/03601270590962523

[24] Savundranayagam MY Montgomery RJ Kosloski K Little TD. Impact of a psychoeducational program on three types of caregiver burden among spouses. Int J Geriatr Psychiatry. (2011) 26(4):388–96. 10.1002/gps.2538 20652873

[25] Braun M Mura K Peter-Wight M Hornung R Scholz U. Toward a better understanding of psychological well-being in dementia caregivers: the link between marital communication and depression. Fam Process. (2010) 49(2):185–203. 10.1111/j.1545-5300.2010.01317.x 20594206

[26] Whitlatch C. Informal caregivers: communication and decision making. Am J Nurs. (2008) 108(9):73–7. 10.1097/01.NAJ.0000336426.65440.87 18797237

[27] Lyons KS Lee CS. The theory of dyadic illness management. J Fam Nurs. (2018) 24(1):8–28. 10.1177/1074840717745669 29353528

[28] Dart RC Surratt HL Cicero TJ Parrino MW Severtson SG Bucher-Bartelson B Trends in opioid analgesic abuse and mortality in the United States. N Engl J Med. (2015) 372(3):241–8. 10.1056/NEJMsa1406143 25587948

[29] Chan AS Ho YC Cheung MC Albert MS Chiu HF Lam LC. Association between mind-body and cardiovascular exercises and memory in older adults. J Am Geriatr Soc. (2005) 53(10):1754–60. 10.1111/j.1532-5415.2005.53513.x 16181176

[30] Kirkwood G Rampes H Tuffrey V Richardson J Pilkington K. Yoga for anxiety: a systematic review of the research evidence. Br J Sports Med. (2005) 39(12):884–91.; discussion 91. 10.1136/bjsm.2005.018069 16306493 PMC1725091

[31] Ross A Thomas S. The health benefits of yoga and exercise: a review of comparison studies. J Altern Complement Med. (2010) 16(1):3–12. 10.1089/acm.2009.0044 20105062

[32] Woodyard C. Exploring the therapeutic effects of yoga and its ability to increase quality of life. Int J Yoga. (2011) 4(2):49–54. 10.4103/0973-6131.85485 22022122 PMC3193654

[33] Patel NK Newstead AH Ferrer RL. The effects of yoga on physical functioning and health related quality of life in older adults: a systematic review and meta-analysis. J Altern Complement Med. (2012) 18(10):902–17. 10.1089/acm.2011.0473 22909385

[34] Schmid AA Van Puymbroeck M Altenburger P Schalk T Miller K Damush T Poststroke balance improves with yoga. Stroke. (2012) 43(9):2402–7. 10.1161/STROKEAHA.112.658211 22836351

[35] Schmid A Miller K Van Puymbroeck M DeBaun E. Yoga leads to multiple physical improvements after stroke. Complement Ther Med. (2014) 22:994–1000. 10.1016/j.ctim.2014.09.005 25453519

[36] Schmid AA Miller KK Van Puymbroeck M Schalk N. Feasibility and results of a case study of yoga to improve physical functioning in people with chronic traumatic brain injury. Disabil Rehabil. (2016) 38(9):914–20. 10.3109/09638288.2015.1062927 26208245

[37] Grimm LA Van Puymbroeck M KKM Fisher T Schmid AA. Yoga after traumatic brain injury: changes in emotional regulation and health-related quality of life in a case- study. Int J Complement Altern Med. (2017) 8(1):00247. 10.15406/ijcam.2017.08.00247

[38] Roney MA Sample PL Stallones L Van Puymbroeck M Schmid AA. The lived experience of individuals with chronic traumatic brain injury: an adapted group yoga intervention. OBM Integr Complement Med. (2018) 3(4):1–19. 10.21926/obm.icm.1804033

[39] Schmid A Miller K Van Puymbroeck M DeBuaun-Sprague E. Yoga improves physical functioning in people with chronic traumatic brain injury: a case-study. Arch Phys Med Rehabil. (2014) 95(10):E51. 10.1016/j.apmr.2014.07.157

[40] Stephens J Van Puymbroeck M Sample P Schmid A. Yoga improves balance, mobility, and perceived occupational performance in adults with chronic brain injury: a preliminary investigation. Complement Ther Clin Pract. (2020) 40:101172. 10.1016/j.ctcp.2020.101172 32347208 PMC7483737

[41] Miller KK Schmid AA. Perceived Benefits and Value of Community-Based Adapted-Yoga for Persons with Acquired Brain Injury. Anaheim: APTA (2016).

[42] Stephens JA Press D Atkins J Duffy JR Thomas ML Weaver JA Feasibility of acquiring neuroimaging data from adults with acquired brain injuries before and after a yoga intervention. Brain Sci. (2023) 13(10):1413. 10.3390/brainsci13101413 37891782 PMC10605412

[43] Stephens JA Hernandez Sarabia J Sharp JL Leach H Bell C Thomas M Adaptive yoga vs low-impact exercise for adults with chronic acquired brain injury: a pilot randomized control trial protocol. Front Hum Neurosci. (2023) 17:1291094. 10.3389/fnhum.2023.1291094 38077184 PMC10701427

[44] Van Puymbroeck M Walter AA Hawkins BL Sharp J Woschkolup K Urrea-Mendoza E Functional improvements in Parkinson’s disease following a randomized trial of yoga. Evidence-Based Complementary Altern Med. (2018) 2018:8516351. 10.1155/2018/8516351 PMC6009016 29967649

[45] Van Puymbroeck M Schmid A Walter A Hawkins B. Improving leisure constraints in older adults with a fear of falling through hatha yoga: an acceptability and feasibility study. Int J Gerontol Geriatr Res. (2017) 1(1):8–13.

[46] Van Puymbroeck M Smith R Schmid AA. Yoga as a means to negotiate physical activity constraints in middle-aged and older adults. Int J Disabil Hum Develop. (2011) 10(2):117–21. 10.1515/ijdhd.2011.029

[47] Schmid AA van Puymbroeck M Koceja DM. Effect of a 12-week yoga intervention on fear of falling and balance in older adults: a pilot study. Arch Phys Med Rehabil. (2010) 91(4):576–83. 10.1016/j.apmr.2009.12.018 20382290

[48] Van Puymbroeck M Burk BN Shinew KJ Kuhlenschmidt MC Schmid AA. Perceived health benefits from yoga among breast cancer survivors. Am J Health Promot. (2013) 27(5):308–15. 10.4278/ajhp.110316-QUAL-119 23402226

[49] Van Puymbroeck M Davitt-Burk B Shinew K Kuhlenschmidt M Schmid A. Yoga as a tool to improve health for breast cancer survivors. Am J Health Promot. (2013) 27(5):308–15. 10.4278/ajhp.110316-QUAL-119 23402226

[50] Van Puymbroeck M Schmid AA Shinew K Hsieh PC. Influence of hatha yoga on the physical fitness, physical activity constraints, and body image of breast cancer survivors: a pilot study. Int J Yoga Therap. (2011) 21:81–92. 10.17761/ijyt.21.1.n852143rv21x188u 22398344

[51] Van Puymbroeck M Payne LL Hsieh PC. A phase I feasibility study of yoga on the physical health and coping of informal caregivers. Evidence-Based Complementary Altern Med. (2007) 4(4):519–29. 10.1093/ecam/nem075 PMC2176147 18227920

[52] Van Puymbroeck M Payne LL Hsieh PC. Physiological benefits of an 8 week yoga program for informal caregivers. Evid Based Complement Altern Med. (2007) 4(4):519–29. 10.1093/ecam/nem075 PMC2176147 18227920

[53] Walter AA Van Puymbroeck M Hawkins BL Woschkolup K Urrea-Mendoza E Revilla F Perceived impacts for caregivers following a yoga intervention for people with Parkinson’s disease. Am J Recreat Ther. (2016) 97(10):e77–8. 10.1016/j.apmr.2016.08.236

[54] Wren AA Wright MA Carson JW Keefe FJ. Yoga for persistent pain: new findings and directions for an ancient practice. Pain. (2011) 152(3):477. 10.1016/j.pain.2010.11.017 21247696 PMC3040510

[55] Pearson N Prosko S Sullivan M Taylor MJ. White paper: yoga therapy and pain—how yoga therapy serves in comprehensive integrative pain management, and how it can do more. Int J Yoga Therap. (2020) 30(1):117–33. 10.17761/2020-D-19-00074 32412808

[56] Büssing A Ostermann T Lüdtke R Michalsen A. Effects of yoga interventions on pain and pain-associated disability: a meta-analysis. J Pain. (2012) 13(1):1–9. 10.1016/j.jpain.2011.10.001 22178433

[57] Schmid AA Fruhauf CA Sharp JL Van Puymbroeck M Bair MJ Portz JD. Yoga for people with chronic pain in a community-based setting: a feasibility and pilot RCT. J Evid Based Integr Med. (2019) 24. 10.1177/2515690X19863763 31394910 PMC6689911

[58] Fruhauf C Schmid AA Van Puymbroeck M. The Most Useful Tools in the Powerful Tools for Caregivers Program. New Orleans: The Gerontological Society of America (2016).

[59] National Institutes of Health. National Pain Strategy: A comprehensive population health-level strategy for pain Washington, D.C.2016. Available online at: https://iprcc.nih.gov/docs/HHSNational_Pain_Strategy.pdf (Accessed June 30, 2022).

[60] Mann EG LeFort S VanDenKerkhof EG. Self-management interventions for chronic pain. Pain Manag. (2013) 3(3):211–22. 10.2217/pmt.13.9 24654764

[61] Lorig K Holman H. Self-management education: history, definition, outcomes, and mechanisms. Ann Behav Med. (2003) 26(1):1–7. 10.1207/S15324796ABM2601_01 12867348

[62] Fox AL Swink LA Prabhu N Fruhauf CA Portz JD Van Puymbroeck M Manual development for a multi-modal, dyadic intervention for persistent pain: a qualitative study. Br J Pain. (2022) 16(5):481–9. 10.1177/20494637221090461 36389010 PMC9644100

[63] Cleeland CS. Measurement of pain by subjective report. In: Chapman CR Loeser JD, editors. Advances in Pain Research and Therapy. 12th ed. New York: Raven Press (1989). p. 391–403.

[64] Callahan CM Unverzagt FW Hui SL Perkins AJ Hendrie HC. Six-item screener to identify cognitive impairment among potential subjects for clinical research. Med Care. (2002) 40(9):771–81. 10.1097/00005650-200209000-00007 12218768

[65] Warburton DE Jamnik VK Bredin SS Gledhill N. The physical activity readiness questionnaire for everyone (PAR-Q+) and electronic physical activity readiness medical examination (ePARmed-X+). Health Fitness J Canada. (2011) 4(2):3–17. 10.14288/hfjc.v4i2.103

[66] Julious SA. Sample size of 12 per group rule of thumb for a pilot study. Pharm Stat. (2005) 4(4):287–91. 10.1002/pst.185

[67] Moore CG Carter RE Nietert PJ Stewart PW. Recommendations for planning pilot studies in clinical and translational research. Clin Transl Sci. (2011) 4(5):332–7. 10.1111/j.1752-8062.2011.00347.x 22029804 PMC3203750

[68] Orsmond GI Cohn ES. The distinctive features of a feasibility study: objectives and guiding questions. OTJR occupation. Participation and Health. (2015) 35(3):169–77. 10.1177/1539449215578649 26594739

[69] Ory MG Ahn S Jiang L Lorig K Ritter P Laurent DD National study of chronic disease self-management: six-month outcome findings. J Aging Health. (2013) 25(7):1258–74. 10.1177/0898264313502531 24029414

[70] He W Kowal P Naidoo N. Trends in Health and Well-Being of the Older Populations in SAGE Countries: 2014–2015. International Population Reports. Washington, DC: US Census Bureau, US Government Printing Office (2018).

[71] Gibson BA Van Puymbroeck M Fruhauf CA Schmid AA Portz JD. Yoga for caregiving dyads experiencing chronic pain: protocol development for merging yoga and self-management to develop skills intervention. Int J Yoga. (2021) 14(3):256. 10.4103/ijoy.ijoy_93_21 35017869 PMC8691446

[72] Brosnan P Nauphal M Tompson MC. Acceptability and feasibility of the online delivery of hatha yoga: a systematic review of the literature. Complement Ther Med. (2021) 60:102742. 10.1016/j.ctim.2021.102742 34144493

[73] Leach HJ Hidde MC Portz JD Van Puymbroeck M Sharp JL Fox AL Matching exercise volume in active control groups for yoga interventions. Altern Ther Health Med. (2022) 29:AT7433–AT. 35687710

[74] Turk DC Dworkin RH Burke LB Gershon R Rothman M Scott J Developing patient-reported outcome measures for pain clinical trials: iMMPACT recommendations. Pain. (2006) 125(3):208–15. 10.1016/j.pain.2006.09.028 17069973

[75] Ware JE Keller SD Kosinski M. SF-12: How to Score the SF-12 Physical and Mental Health Summary Scales. Health Institute, New England Medical Center (1995).

[76] Deyo R Katrina Ramsey D Buckley L Michaels A Kobus E Eckstrom V Performance of a patient reported outcomes measurement information system (PROMIS) short form in older adults with chronic musculoskeletal pain. Pain Med. (2016) 17(2):314–24. 10.1093/pm/pnv046 26814279 PMC6281027

[77] Law M Baptiste S McColl M Opzoomer A Polatajko H Pollock N. The Canadian occupational performance measure: an outcome measure for occupational therapy. Can J Occup Ther. (1990) 57(2):82–7. 10.1177/000841749005700207 10104738

[78] Schmid AA Van Puymbroeck M Fruhauf CA Bair MJ Portz JD. Yoga improves occupational performance, depression, and daily activities for people with chronic pain. Work. (2019) 63(2):181–9. 10.3233/WOR-192919 31156199

[79] Carpenter L Baker GA Tyldesley B. The use of the Canadian occupational performance measure as an outcome of a pain management program. Can J Occup Ther. (2001) 68(1):16–22. 10.1177/000841740106800102 11233684

[80] NIH. National Center for Complementary and Integrative Health. Pilot Studies: Common Uses and Misuses. (2023). Available online at: https://www.nccih.nih.gov/grants/pilot-studies-common-uses-and-misuses#:∼:text=The%20goal%20of%20pilot%20studies,size%20of%20your%20pilot%20study (cited 2023).

[81] McCarthy MJ Sanchez A Garcia YE Lyons KS Bakas T. Feasibility of the hand in hand relationship intervention for stroke survivor–caregiver dyads: a randomized trial. Res Soc Work Pract. (2021) 31(1):75–89. 10.1177/1049731520961172

[82] Agha Z Schapira RM Laud PW McNutt G Roter DL. Patient satisfaction with physician–patient communication during telemedicine. Telemed J E Health. (2009) 15(9):830–9. 10.1089/tmj.2009.0030 19919189

[83] Harrison M Fullwood C Bower P Kennedy A Rogers A Reeves D. Exploring the mechanisms of change in the chronic disease self-management programme: secondary analysis of data from a randomised controlled trial. Patient Educ Couns. (2011) 85(2):e39–47. 10.1016/j.pec.2010.10.026 21112719

[84] Turmel D Carlier S Bruyneel AV Bruyneel M. Tailored individual yoga practice improves sleep quality, fatigue, anxiety, and depression in chronic insomnia disorder. BMC Psychiatry. (2022) 22(1):267. 10.1186/s12888-022-03936-w 35421962 PMC9012014

[85] Khoshrounejad F Hamednia M Mehrjerd A Pichaghsaz S Jamalirad H Sargolzaei M Telehealth-based services during the COVID-19 pandemic: a systematic review of features and challenges. Front Public Health. (2021) 977. 10.3389/fpubh.2021.711762 PMC8326459 34350154

[86] Wootton AR McCuistian C Legnitto Packard DA Gruber VA Saberi P. Overcoming technological challenges: lessons learned from a telehealth counseling study. TelemedJ E Health. (2020) 26(10):1278–83. 10.1089/tmj.2019.0191 31800368 PMC11708184

[87] Jhaveri K Cohen JA Barulich M Levin AO Goyal N Loveday T Soup cans, brooms, and zoom:” rapid conversion of a cancer survivorship program to telehealth during COVID-19. Psychooncology. (2020) 29(9):1424–6. 10.1002/pon.5473 32672845 PMC7405495

